# Dr. Ybarra’s Article on Yellow Fever

**Published:** 1907-10

**Authors:** 


Dr. Ybarra's Article on Yellow Fever.—It would seem late in the day to question the rôle of the mosquito in the spread of yellow fever, for it is almost universally admitted that the mosquito is the *only known* factor in its dissemination. But that there may be and doubtless are other methods of its spread as yet unknown or unsuspected is highly probable. Many experienced yellow fever physicians have seen first cases occur under circumstances which seemed to preclude the possibility of mosquito agency. I have often related my own observations and cited such cases. Professor Bemis, in Zeimsen's *System of Medicine,* recounts such cases; Dr. Carter, of the M. H. S., relates similar cases, citing especially the cases at Clinton, Miss., the year that yellow fever spread from the dengue epidemic at Ocean Springs, Miss. (1899), to Edwards, thence to towns in the interior. The literature of the subject is full of them. These observations long puzzled the profession, and an "atmospheric condition of infection" was hypothecated and accepted, "fomites," carriers of infection, until the discovery of the fact that the *stegomyia* is the intermediary host. Then all other idea of how the disease is spread was rashly abandoned, and precautions against the deadly *steg. fasc.,* or, more recently, the *steg. callopus,* alone are taken.

Dr. Ybarra's observations, which are published herewith, a valuable contribution to the record, bear out my (and his) contention that there my be other means of spread as yet unknown, and that cases of yellow fever *may* occur quite independently of the mosquito. While the experiments of Read, Carroll and Agramonte, so often detailed in the medical press, abundantly prove that yellow fever soiled garments and bedding will not communicate the disease *by contact,* and even the swallowing of black vomit does not infect, it has not been proved that the feces, urine and vomit do not contain the infective principle, and that it *may be* communicated by the filth-loving stable fly, for instance, an insect equipped for stinging. May he not inoculate a person with the poison sucked, say, from yellow fever feces, as it is believed that he may and does inoculate with the typhoid or the diphtheria bacillus?

We do not know all about yellow fever yet, or of anything, in fact. It is a very recent discovery that a person who had typhoid fever and recovered years ago may be a carrier of the infection still, and may infect whole families years after having himself recovered. The case of the Vienese bakeress, related by Metchinkoff (Harbin Lectures), and the cases more recently observed and reported by Stiles, in Washington, are illustrations.

The wise physician called to deal with yellow fever will, therefore, take precautions against a spread of the disease other than screening the room. The excreta should be promptly destroyed, and none but immunes should be permitted to come in touch with the patient. I commend Dr. Ybarra's paper to the thoughtful consideration of my readers. I think dengue and yellow fever are degrees of *the same disease*, the former modified in some way as yet unknown. Precautions should be taken against dengue the same as against yellow fever.

